# Demographic characteristics of transfusion-transmitted infections among blood donors in China

**DOI:** 10.1186/s12879-019-4044-x

**Published:** 2019-06-11

**Authors:** Le Chang, Junpeng Zhao, Fei Guo, Huimin Ji, Lu Zhang, Xinyi Jiang, Lunan Wang

**Affiliations:** 1 National Center for Clinical Laboratories, Beijing Hospital, National Center of Gerontology, Beijing, People’s Republic of China; 2 Beijing Engineering Research Center of Laboratory Medicine, Beijing Hospital, Beijing, People’s Republic of China; 3 Graduate School, Peking Union Medical College, Chinese Academy of Medical Sciences, Beijing, People’s Republic of China

**Keywords:** Transfusion-transmitted infections, Demographic characteristics, Human immunodeficiency virus, Hepatitis B virus, Hepatitis C virus, *Treponema pallidum*

## Abstract

**Background:**

Demographic characteristic surveillance of transfusion-transmitted infections (TTIs) among blood donors is crucial to formulating control strategies and preventing TTIs. This study aimed to investigate the demographic characteristics and social factors associated with TTIs among blood donors from 14 different blood centers or banks in China, covering almost the entire China.

**Methods:**

Demographic information of 1976 blood donations were obtained from the donor databases of 14 blood centers. The results of the samples were confirmed by the National Center for Clinical Laboratories (NCCL).

**Results:**

Of the 1976 donations, 928 were confirmed as TTI positive (HBV, 309; HCV, 162; HIV, 116; syphilis, 341), while 1048 tested negative. The differences in demographic distribution of TTI positive and negative donations regarding age, previous donation history, occupation, and education were statistically significant (*p* < 0.001). The factors mentioned above and marital status had associations with TTIs. Among the TTIs, only syphilis was related to ethnicity (adjusted odds ratio [aOR]: 2.309, 95% confidence interval [CI]: 1.378–3.868, *p* = 0.001), and only HBV positivity was not associated with marital status (HBV, aOR: 0.933, 95% CI: 0.670–1.299, *p* = 0.681). Gender and education were independent predictors of HIV and syphilis infections (*p* < 0.05).

**Conclusions:**

Demographic characteristics in this study included age, gender, previous donation history, ethnicity, marital status, occupation, and education, some of which were associated with TTIs. The most susceptible populations for TTIs were unmarried males and first-time donors aged between 26 and 55 years, and blood donors who were workers or company employees with low-educational level. Timely surveillance and updated demographic data on blood donors are critical for blood safety.

## Background

Transfusion-transmitted infections (TTIs), principally hepatitis B virus (HBV), hepatitis C virus (HCV), human immunodeficiency virus (HIV), and syphilis caused by *Treponema pallidum* (TP) among blood donors remain a major threat to blood safety [1]. The World Health Organization recommends that all blood donors be screened for HBV, HCV, HIV, and TP. Hence, comprehensive surveillance and control of TTIs among blood donors is essential to ensuring the strict safety of blood supply, particularly with the dramatic change in prevalence of TTIs in China over the past 10 years [2]. Obtaining more demographic data regarding TTIs from volunteer blood donors, who comprise the general population, provides a scientific basis for formulating control strategies and measures towards these groups, and plays a critical role in preventing unidentified infectious diseases from getting into the blood supply chain and predicting the future domestic course of TTIs. Currently, there are limited comprehensive data about the demographic characteristics among blood donors regarding major TTIs (HBV, HCV, HIV, and TP) in China. Therefore, the present study aimed to investigate the demographic characteristics and social factors associated with TTIs among blood donors from 14 different blood centers or banks in China, covering almost the entire China.

## Methods

The study protocol was approved by the ethics committee of the NCCL. Written informed consent at the time of blood donation was obtained from all blood donors participating in this study.

### Study samples

During March 2015 and September 2015, 3719 blood donations collected from 14 different blood centers or blood banks (Table 1) including Changchun (abbreviated as CC), Chongqing (CQ), Hebei (HB), Heilongjiang (HLJ), Henan (HN), Heze (HZ), Jiangsu (JS), Liaoning (LN), Shandong (SD), Shenzhen (SZ), Tianjin (TJ), Tongzhou (TZ), Yunnan (YN), and Xiangyang (XY) were screened for TTIs (hepatitis B surface antigen [HBsAg], anti-HCV, anti-HIV, and anti-TP) using one or two screening enzyme-linked immunosorbent assays (ELISAs) of each serologic marker. The blood donations that tested serologically reactive for TTIs or serologically non-reactive for TTIs (elevated alanine aminotransferase [ALT] level > 50 U/L) were not transfused and enrolled in this study. These samples that tested initially reactive for TTIs were sent to the NCCL for retesting. Meanwhile, demographic data were obtained from the donor/donation database of each blood center or blood bank.

**Table 1:** Stations of study participation and their commercially available HIV serology kits

Province	Stations	Abbreviations of TTI kits			
		HBV	HCV	HIV	TP
Jilin	CC				
Chongqing	CQ				
Heilongjiang	HLJ				
Hebei	HB	KHB-ELISA	KHB-ELISA	KHB-ELISA	
Henan	HN	Intec-ELISA	Intec-ELISA	Intec-ELISA	KHB-ELISA
Shandong	SD, HZ	Livzon-ELISA	Livzon-ELISA	Livzon-ELISA	Intec-ELISA
Guangdong	SZ	Murex-ELISA	Murex-ELISA	Livzon-ELISA 4^th^	Livzon-ELISA
Tianjin	TJ	Ortho-ELISA	Ortho-ELISA	Murex-ELISA 4^th^	Wantai-ELISA
Hubei	XY	Wantai-ELISA	Wantai-ELISA	Wantai-ELISA	Murex-ELISA
Jiangsu	JS	Mérieux-ELISA		Wantai-ELISA 4^th^	
Beijing	TZ				
Liaoning	LN				
Yunnan	YN				
		ABBOTT-CLIA	ABBOTT-CLIA	Roche CTM/MPX 2.0	ABBOTT-CLIA
		Roche-ECLIA	Roche-ECLIA	MP Western Blot	Roche-ECLIA
		Roche CTM/MPX 2.0	Roche CTM/MPX 2.0		Fujirebio-TPPA
Beijing	NCCL	ABBOTT HBsAg Confirmatory Test	Mikrogen RIBA		Mikrogen RIBA-IgG
		Roche HBsAg Confirmatory Test			Mikrogen RIBA-IgM

1. HBsAg: KHB Diagnostic Kit for Hepatitis B Virus Surface Antigen (Shanghai Kehua Bioengineering co., Shanghai, China); InTec Diagnostic Kit for Hepatitis B Virus Surface Antigen (InTec Products, Inc., Xiamen, China), Livzon Diagnostic Kit for Hepatitis B Virus Surface Antigen (Zhuhai Livzon Diagnostics Inc., Zhuhai, China), Murex HBsAg Version 3 (Diasorin, Saluggia, Italy), Ortho HBsAg ELISA Test System 3 (Ortho-Clinical Diagnostics, Raritan, New Jersey), Wantai Diagnostic Kit for Hepatitis B Virus Surface Antigen (Beijing Wantai Biological Pharmacy, Beijing, China). ABBOTT PRISM HBsAg and Confirmatory test (Abbott Diagnostics, Abbott Park, IL, US) , Roche HBsAg II and Confirmatory test (Roche Diagnostics GmbH, Penzberg, Germany)

2. Anti-HCV: KHB Diagnostic Kit for Antibody to Hepatitis C virus (Shanghai Kehua Bioengineering co., Shanghai, China), InTec Diagnostic Kit for Antibody to Hepatitis C virus (InTec Products, Inc., Xiamen, China), Livzon Diagnostic Kit for Antibody to Hepatitis C virus (Zhuhai Livzon Diagnostics Inc., Zhuhai, China), Murex anti-HCV (version 4.0) (Diasorin, Saluggia, Italy), ORTHO HCV Version 3.0 ELISA (Ortho-Clinical Diagnostics, Raritan, New Jersey), Wantai Diagnostic Kit for Antibody to Hepatitis C virus (Beijing Wantai Biological Pharmacy, Beijing, China), ARCHITECT anti-HCV (Abbott Diagnostics, Abbott Park, Illinois, US) , Elecsys Anti-HCV II (Roche Diagnostics GmbH, Penzberg, Germany)

3. Anti-HIV: KHB Diagnostic Kit for Antibody to human immunodeficiency virus (Shanghai Kehua Bioengineering co., Shanghai, China), InTec Diagnostic Kit for Antibody to human immunodeficiency virus (InTec Products, Inc., Xiamen, China), Livzon Diagnostic Kit for Antibody to human immunodeficiency virus (Zhuhai Livzon Diagnostics Inc., Zhuhai, China), Livzon Diagnostic Kit for Antibody and Antigen to human immunodeficiency virus (4^th^ generation) (Zhuhai Livzon Diagnostics Inc., Zhuhai, China), Murex HIV Ag/Ab Combination (4^th^ generation) (Diasorin, Saluggia, Italy), Wantai Diagnostic Kit for Antibody to human immunodeficiency virus (Beijing Wantai Biological Pharmacy, Beijing, China), Wantai Diagnostic Kit for Antibody and Antigen to human immunodeficiency virus (4^th^ generation) (Beijing Wantai Biological Pharmacy, Beijing, China)

4. Anti-TP: KHB Diagnostic Kit for Antibody to Treponema Pallidum (Shanghai Kehua Bioengineering co., Shanghai, China), InTec Diagnostic Kit for Antibody to Treponema Pallidum (InTec Products, Inc., Xiamen, China), Livzon Diagnostic Kit for Antibody to Treponema Pallidum (Zhuhai Livzon Diagnostics Inc., Zhuhai, China), Wantai Diagnostic Kit for Antibody to Treponema Pallidum (Beijing Wantai Biological Pharmacy, Beijing, China), Murex Diagnostic Kit for Antibody to Treponema Pallidum (Diasorin, Saluggia, Italy), ARCHITECT syphilis TP system (Abbott, Wiesbaden, Germany), Elecsys Syphilis (Roche Diagnostics, Mannheim, Germany)

### TTI screening and confirmatory algorithms

**Fig. 1:**
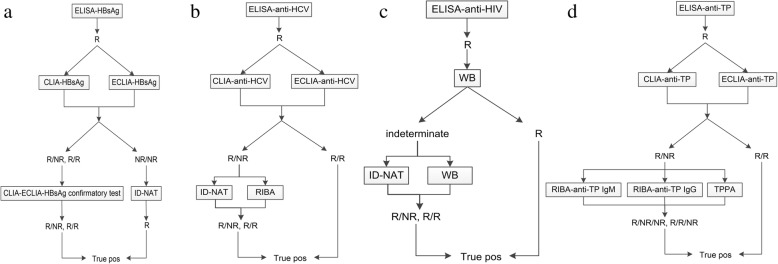
TTIs confirmatory algorithms. R: reactive. NR: non-reactive. Pos: Positive

Serological screening assays for each marker were performed on all donations at every blood bank using at least one ELISA kit (Table 1). All samples that tested serologically non-reactive for HBV, HCV, and HIV were confirmed by nucleic acid testing at every blood center or blood bank. All blood donations that tested serologically reactive for TTIs (HBsAg, anti-HCV, anti-HIV, and anti-TP) underwent further confirmatory testing at the NCCL (Fig. 1).

### Statistical analyses

True positive tests for TTIs and the samples that tested non-reactive for TTIs (ALT level > 50 U/L) were included in the statistical analysis using SPSS version 21.0 (IBM Corp., Armonk, NY, USA). Chi-square tests were performed on all examined outcomes by categorizing donor demographic factors. *P* < 0.05 was considered statistically significant. The correlation of dichotomized factor scores with TTIs was examined by bivariate logistic regression analysis. Additionally, multivariate logistic regression analysis was used to investigate the role of individual demographic factors on the outcome of TTI status.

## Results

### Confirmatory results of plasma samples

Of 3719 plasma samples, 2671 were initially tested reactive for TTIs at 14 blood centers or blood banks (HBsAg, 896; anti-HCV, 582; anti-HIV, 400; anti-TP, 793). Among the 2671 initially reactive samples for TTIs, 928 were confirmed as positive (HBV, 309; HCV, 162; HIV, 116; TP, 341); 1048 samples that tested non-reactive for TTIs (HBsAg, anti-HCV, anti-HIV, and anti-TP), but discarded due to ALT level > 50 U/L, were confirmed as negative for TTIs. Therefore, the demographic characteristics of 928 blood donors with TTIs and 1048 blood donors without TTIs were analyzed in this study.

### Demographic characteristics

The demographic information of 1976 donors is shown in Table 2. The majority of donors were younger than 45 years (85.9%, 1697/1976). There were more men than women (68.5%, 1354/1976 vs 31.5%, 622/1976), and only 5.1% (101/1976) were non-Han minority donors. Meanwhile, more than half were married (54.3%, 1073/1976) and first-time donors (78.0%, 1542/1976) among all donors with low education level (secondary school or below: 60.3%, 1192/1976). Workers (16.8%, 332/1976) and company employees (13.3%, 262/1976) were two major donation sources for the study groups, after the removal of the “others” category. The distribution of gender (*p* = 0.991), ethnicity (*p* = 0.080), and marital status (*p* = 0.362) in the overall TTI population showed no significant differences with the negative groups. However, more older (age ≥ 36 years, positive: 45.9% [426/928] vs negative: 33.3% [349/1048], *p* < 0.001) donors with less possibility of donating blood repeatedly (repeat donor, positive: 11.4% [106/928] vs negative: 31.3% [328/1048], *p* < 0.001) and with lower education level (secondary school or below, positive: 65.7% [610/928] vs negative: 55.5% [582/1048], *p* < 0.001) tested positive for TTIs than the negative group. Furthermore, the distribution of occupation among TTI positive donors significantly differed from negative donors (*p* < 0.001).

**Table 2:** Demographic characteristics distribution of blood donors by TTIs status^*^

Characteristics	HBV	HCV	HIV	Syphilis (TP)	TTIs	Non-reactive for TTIs	Sum
	*n*=309	*n*=162	*n*=116	*n*=341	*n*=928	*n*=1048	*n*=1976
Age (years old)							
18–25	71 (3.6%)	48 (2.4%)	47 (2.4%)	62 (3.1%)	228 (11.5%)	369 (18.7%)	597 (30.2%)
26–35	90 (4.6%)	36 (1.8%)	45 (2.3%)	103 (5.2%)	274 (13.9%)	330 (1.7%)	307 (15.5%)
36–45	88 (4.5%)	44 (2.2%)	17 (0.9%)	121 (6.1%)	270 (13.7%)	226 (11.4%)	496 (25.1%)
46–55	59 (3.0%)	33 (1.7%)	6 (0.3%)	53 (2.7%)	151(7.6%)	117 (5.9%)	268 (13.6%)
>55	1 (0.1%)	1 (0.1%)	1 (0.1%)	2 (0.1%)	5 (0.3%)	6 (0.3%)	11 (0.6%)
Gender							
Female	87 (4.4%)	47 (2.4%)	5 (0.3%)	153 (7.7%)	292 (14.8%)	330 (16.7%)	622 (31.5%)
Male	222 (11.2%)	115 (5.8%)	111 (5.6%)	188 (9.5%)	636 (32.2%)	718 (36.3%)	1354 (68.5%)
Previous donation history							
Repeat donor	9 (0.5%)	12 (0.6%)	37 (1.9%)	48 (2.4%)	106 (5.4%)	328 (16.6%)	434 (22.0%)
First-time donor	300 (15.2%)	150 (7.6%)	79 (4.0%)	293 (14.8%)	822 (41.6%)	720 (36.4%)	1542 (78.0%)
Ethnicity							
Minority	13 (0.7%)	9 (0.5%)	5 (0.3%)	29 (1.5%)	56 (2.8%)	45 (2.3%)	101 (5.1%)
Han	296 (15.0%)	153 (7.7%)	111 (5.6%)	312 (15.8%)	872 (44.1%)	1003 (50.8%)	1875 (94.9%)
Marital status							
Married	184 (9.3%)	80 (4.0%)	42 (2.1%)	208 (10.5%)	514 (26.0%)	559 (28.3%)	1073 (54.3%)
Unmarried	125 (6.3%)	82 (4.1%)	74 (3.7%)	133 (6.7%)	414 (21.0%)	489 (24.7%)	903 (45.7%)
Occupation							
Teacher/civil servant/medical worker	3 (0.2%)	2 (0.1%)	2 (0.1%)	5 (0.3%)	12 (0.6%)	46 (2.3%)	58 (2.9%)
Farmer	27 (1.4%)	12 (0.6%)	2 (0.1%)	20 (1.0%)	61 (3.1%)	43 (2.2%)	104 (5.3%)
Student	31 (1.6%)	13 (0.7%)	16 (0.8%)	17 (0.9%)	77 (3.9%)	119 (6.0%)	196 (9.9%)
Worker	60 (3.0%)	30 (1.5%)	36 (1.8%)	51 (2.6%)	177 (9.0%)	155 (7.8%)	332 (16.8%)
Company employee	48 (2.4%)	20 (1.0%)	24 (1.2%)	37 (1.9%)	129 (6.5%)	133 (6.7%)	262 (16.8%)
Others	140 (7.1%)	85 (4.3%)	36 (1.8%)	211 (10.7%)	472 (23.9%)	552 (27.9%)	1024 (51.8%)
Education							
Masters/Bachelor degree	54 (2.7%)	29 (1.5%)	21 (1.1%)	48 (2.4%)	152 (7.7%)	270 (13.7%)	422 (21.4%)
Associate degree	51 (2.6%)	27 (1.4%)	41 (2.1% )	47 (2.4%)	166 (8.4%)	196 (9.9%)	362 (18.3%)
Secondary school or below	204 (10.3%)	106 (5.4%)	54 (2.7%)	246 (12.4%)	610 (30.9%)	582 (29.5%)	1192 (60.3%)

^*^:%=n/(928+1048)*100%

**Fig. 2:**
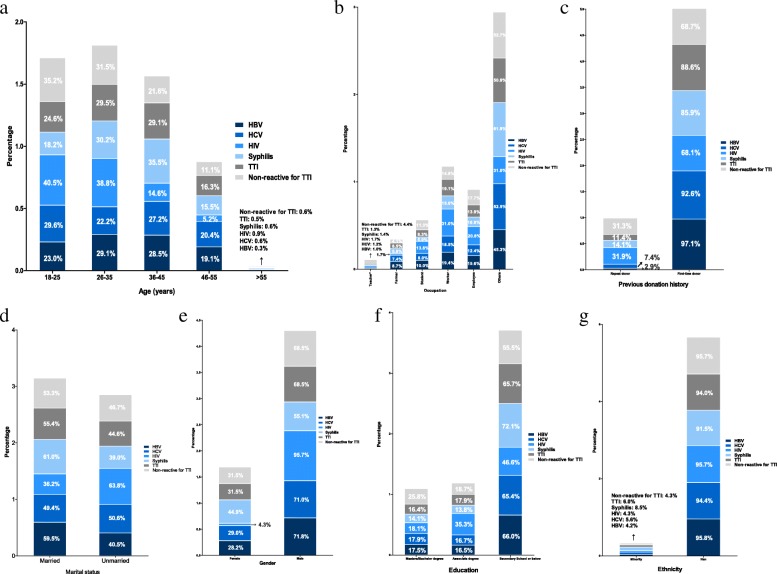
The discrepancies of demographic distribution among every TTI. Teacher*: Teacher/civil servant/medical worker

In comparison to the overall TTI sample, the demographic data of the HCV, HIV, and TP positive groups had special characteristics (Fig. 2). Only the demographic information of HBV positive populations was basically consistent with the overall TTI populations. In the HCV positive group, the overall TTI positive group differed from the negative group in that the occupation of donors with HCV infection showed no significant difference in the negative groups (*p* = 0.087). In the HIV positive groups, the proportion of unmarried donors (HIV positive: 63.8% [74/116] vs negative: 46.7% [489/1048], *p* < 0.001), male donors (HIV positive: 95.7% [111/116] vs negative: 68.5% [718/1048], *p* < 0.001), who were workers (HIV positive: 31.0% [36/116] vs negative: 14.8% [155/1048], *p* < 0.001), with associate degrees (HIV positive: 35.3% [41/116] vs negative: 18.7% [196/1048], *p* < 0.001) were apparently higher than negative groups, while there was no significant differences in the distribution of age, previous donation history, and ethnicity among HIV positive individuals and TTI negative individuals. Besides, only the marital status of HIV positive groups among all kinds of TTIs showed a significant difference with the populations with non-reactivity for TTIs. Specifically, all the socio-demographic factors of TP positive subjects were significantly different from those of TTI negative subjects (*p* < 0.05).

Apart from the differences between each TTI and the negative samples, discrepancies in the demographic distribution for each TTI were also found. As shown in Fig. 2-a, in the age groups of 18–25 and 26–35 years for all TTIs, HIV positive populations occupied the highest proportion (age [18–25]: 40.5%, 47/116; age [26–35]: 38.5%, 45/116). Among the 36–45 and 46–55 year age groups for each TTI, syphilis (age [36–45]: 35.5%, 121/341) and HCV (age [46–55]: 20.4%, 33/162) positive subjects showed the higher proportions, respectively. As shown in Fig. 2-e, for each TTI gender group, the proportion of females with syphilis (44.9%) was higher than those with HBV, HCV, and HIV, while among male donors with each TTI, blood donors with HIV infection showed the highest proportion (95.7%, 111/116). Regarding previous donation history, HIV positive populations showed more previous donation histories than populations with other TTIs (HIV: 31.9%, 37/116; HBV: 2.9%, 9/309; HCV: 7.4%, 12/162; and syphilis: 14.1%, 48/341) (Fig. 2-c). According to data regarding ethnicity and marital status (Table 2), only the groups with TP positivity (ethnicity, TP positive: 8.5% [29/341] vs negative: 4.3% [45/1048]) and HIV positivity (married, HIV positive: 36.2% [42/116] vs negative: 53.3% [559/1048]) showed significant differences in the groups that tested non-reactive for TTIs, respectively. Therefore, among the minority and unmarried groups of each TTI, TP and HIV positive subjects had the highest proportions, respectively (Fig. 2-g, d). As shown in Fig. 2-b higher proportions of HIV positive individuals were students (13.8%, 16/116), workers (31.0%, 36/116), and company employees (20.8%, 24/116) compared to other TTIs.

### Demographic factors associated with TTIs

A logistic regression model was used to investigate the correlation of demographic features of subjects with the results of TTIs status. Demographic characteristics of the overall TTI population including age, previous donation history, marital status, occupation, and education associated with TTIs status were identified through the bivariate analyses in the logistic regression models (*p* < 0.05) (Table 3). Differences were found in each TTI after bivariate analysis. HIV, subjects who tested positive for the other three TTIs showed significant differences compared to those who tested negative regarding age and previous donation history in the logistic regression models (*p* < 0.05) (Tables 4 and 5). Among all TTIs, the gender group of HIV (male, odds ratio [OR]: 9.652, 95% CI: 3.863–24.115, *p* < 0.001) and syphilis (male, OR: 0.532, 95% CI: 0.406–0.697, *p* < 0.001) positive individuals were significantly different from negative groups as analyzed by logistic regression models, unlike the HBV and HCV positive individuals (Table 5). Besides, marital status had no relationship with HBV infection (OR: 0.945, 95% CI: 0.660–1.354, *p* = 0.758), but was associated with HCV, HIV, and syphilis. In the occupation groups among TTIs, only HCV positive subjects did not significantly differ from the negative population (*p* > 0.05) (Table 4). Education level was a predictor of HIV (associate degree, OR: 1.884, 95% CI: 1.168–3.037, *p* = 0.009) and syphilis (masters/bachelor degree, OR: 0.596, 95% CI: 0.398–0.892, *p* = 0.012) positivity.

**Table 3:** Logistic regression predicting TTIs positive status

Demographic factors	TTIs			
	Bivariate analysis		Multivariable analysis	
	OR (95% CI)	*p*-value	aOR (95% CI)	*p*-value
Age				
>55 (Reference)	1	–	1	–
18–25	2.690 (0.724–10.000)	0.140	2.616 (0.705–9.713)	0.151
26–35	2.874 (1.982–4.167)	<0.001	2.799 (1.948–4.023)	<0.001
36–45	2.611 (1.879–3.627)	<0.001	2.562 (1.862–3.525)	<0.001
46–55	1.802 (1.351–2.404)	<0.001	1.813 (1.376–2.389)	<0.001
Gender				
Female (Reference)	1	–	1	–
Male	0.994 (0.810–1.219)	0.951	1.041 (0.852–1.271)	0.697
Previous donation history				
First-time donor (Reference)	1	–	1	–
Repeat donor	0.260 (0.203–0.334)	<0.001	0.261 (0.204–0.334)	<0.001
Ethnicity				
Han (Reference)	1	–	1	–
Minority	1.480 (0.963–2.273)	0.074	1.489 (0.974–2.276)	0.066
Marital status				
Unmarried (Reference)	1	–	1	–
Married	0.681 (0.533–0.870)	0.002	0.720 (0.573–0.904)	0.005
Occupation				
Others (Reference)	1	–	1	–
Teacher/civil servant/medical worker	0.350 (0.179–0.684)	0.002	0.355 (0.181–0.694)	0.002
Farmer	1.176 (0.761–1.817)	0.465	1.191 (0.772–1.838)	0.430
Student	1.119 (0.769–1.628)	0.556	1.000 (0.695–1.438)	1.000
Worker	1.187 (0.908–1.552)	0.209	1.227 (0.940–1.601)	0.133
Company employee	1.208 (0.901–1.620)	0.206	1.256 (0.940–1.678)	0.123
Education				
Secondary School or below (Reference)	1	–	1	–
Masters/Bachelor degree	0.643 (0.488–0.848)	0.002	0.602 (0.467–0.775)	<0.001
Associate degree	1.007 (0.776–1.307	0.957	0.992 (0.770–1.277)	0.947

**Table 4:** Logistic regression predicting HBV and HCV positive status

Demographic factors	HBV				HCV			
	Bivariate analysis		Multivariable analysis		Bivariate analysis		Multivariable analysis	
	OR (95% CI)	*p*-value	aOR (95% CI)	*p*-value	OR (95% CI)	*p*-value	aOR (95% CI)	*p*-value
Age								
>55 (Reference)	1	–	1	–	1	–	1	–
18–25	3.279 (0.286–37.613)	0.340	2.989 (0.257–34.717)	0.381	4.096 (0.405–41.425)	0.232	4.455 (0.438–45.266)	0.207
26–35	3.735 (2.182–6.393)	<0.001	3.500 (2.093–5.855)	<0.001	3.650 (1.936–6.881)	<0.001	3.818 (2.061–7.072)	<0.001
36–45	2.719 (1.660–4.453)	<0.001	2.638 (1.647–4.227)	<0.001	2.362 (1.321–4.222)	0.004	2.564 (1.465–4.486)	0.001
46–55	1.877 (1.202–2.931)	0.006	1.868 (1.232–2.834)	0.003	1.224 (0.713–2.102)	0.464	1.302 (0.777–2.180)	0.316
Gender								
Female (Reference)	1	–	1	–	1	–	1	–
Male	1.126 (0.832–1.525)	0.442	1.126 (0.836–1.517)	0.433	1.135 (0.776–1.660)	0.513	1.131 (0.777–1.647)	0.521
Previous donation history								
First-time donor (Reference)	1	–	1	–	1	–	1	–
Repeat donor	0.057 (0.029–0.113)	<0.001	0.057 (0.029–0.133)	<0.001	0.162 (0.088–0.300)	<0.001	0.159 (0.087–0.294)	<0.001
Ethnicity								
Han (Reference)	1	–	1	–	1	–	1	–
Minority	0.892 (0.453–1.755)	0.741	0.961 (0.493–1.873)	0.907	1.336 (0.617–2.891)	0.463	1.338 (0.624–2.869)	0.455
Marital status								
Unmarried (Reference)	1	–	1	–	1	–	1	–
Married	0.945 (0.660–1.354)	0.758	0.933 (0.670–1.299)	0.681	0.551 (0.351–0.866)	0.010	0.515 (0.338–0.785)	0.002
Occupation								
Others (Reference)	1	–	1	–	1	–	1	–
Teacher/civil servant/medical worker	0.276 (0.082–0.934)	0.039	0.276 (0.082–0.932)	0.038	0.348 (0.081–1.493)	0.155	0.343 (0.080–1.471)	0.150
Farmer	1.720 (0.976–3.032)	0.061	1.748 (0.993–3.078)	0.053	1.388 (0.672–2.867)	0.375	1.377 (0.672–2.824)	0.382
Student	1.793 (1.037–3.098)	0.036	1.634 (0.966–2.764)	0.067	0.835 (0.405–1.723)	0.626	0.869 (0.430–1.756)	0.695
Worker	1.375 (0.934–2.025)	0.106	1.417 (0.965–2.080)	0.075	1.042 (0.639–1.698)	0.870	1.030 (0.633–1.674)	0.906
Company employee	1.677 (1.101–2.555)	0.016	1.743 (1.150–2.640)	0.009	1.021 (0.587–1.775)	0.942	1.012 (0.586–1.750)	0.965
Education								
Secondary School or below (Reference)	1	–	1	–	1	–	1	–
Masters/Bachelor degree	0.727 (0.481–1.098)	0.129	0.705 (0.487–1.023)	0.066	0.717 (0.428–1.201)	0.206	0.645 (0.402–1.035)	0.069
Associate degree	0.988 (0.668–1.462)	0.952	1.004 (0.689–1.464)	0.983	0.989 (0.606–1.615)	0.966	0.931 (0.577–1.502)	0.769

**Table 5:** Logistic regression predicting HIV and Syphilis positive status

Demographic factors	HIV				Syphilis (TP)			
	Bivariate analysis		Multivariable analysis		Bivariate analysis		Multivariable analysis	
	OR (95% CI)	*p*-value	aOR (95% CI)	*p*-value	OR (95% CI)	*p*-value	aOR (95% CI)	*p*-value
Age								
>55 (Reference)	1	–	1	–	1	–	1	–
18–25	2.680 (0.284–25.336)	0.390	1.625 (0.176–14.989)	0.668	3.843 (0.648–22.804)	0.138	3.879 (0.645–23.334)	0.139
26–35	0.853 (0.311–2.341)	0.757	0.749 (0.281–1.999)	0.564	3.714 (2.174–6.344)	<0.001	3.784 (2.245–6.377)	<0.001
36–45	1.044 (0.504–2.165)	0.907	0.870 (0.429–1.763)	0.698	4.479 (2.798–7.171)	<0.001	4.576 (2.905–7.208)	<0.001
46–55	1.329 (0.774–2.280)	0.302	1.316 (0.791–2.187)	0.290	2.647 (1.721–4.072)	<0.001	2.652 (1.757–4.002)	<0.001
Gender								
Female (Reference)	1	–	1	–	1	–	1	–
Male	9.652 (3.863–24.115)	<0.001	10.393 (4.193–25.762)	<0.001	0.532 (0.406–0.697)	<0.001	0.565 (0.434–0.736)	<0.001
Previous donation history								
First-time donor (Reference)	1	–	1	–	1	–	1	–
Repeat donor	1.056 (0.677–1.646)	0.811	1.149 (0.749–1.762)	0.525	0.309 (0.219–0.437)	<0.001	0.306 (0.216–0.432)	<0.001
Ethnicity								
Han (Reference)	1	–	1	–	1	–	1	–
Minority	1.146 (0.421–3.116)	0.790	0.906 (0.342–2.401)	0.843	2.406 (1.421–4.072)	0.001	2.309 (1.378–3.868)	0.001
Marital status								
Unmarried (Reference)	1	–	1	–	1	–	1	–
Married	0.528 (0.315–0.884)	0.015	0.499 (0.300–0.830)	0.007	0.623 (0.443–0.876)	0.006	0.764 (0.554–1.053)	0.100
Occupation								
Others (Reference)	1	–	1	–	1	–	1	–
Teacher/civil servant/medical worker	0.598 (0.135–2.641)	0.497	0.608 (0.139–2.661)	0.509	0.357 (0.135–0.941)	0.037	0.362 (0.138–0.950)	0.039
Farmer	0.653 (0.148–2.888)	0.575	0.635 (0.145–2.788)	0.547	0.793 (0.436–1.443)	0.448	0.838 (0.462–1.521)	0.562
Student	2.587 (1.203–5.567)	0.015	1.878 (0.909–3.882)	0.089	0.653 (0.351–1.214)	0.178	0.532 (0.293–0.968)	0.039
Worker	2.598 (1.539–4.374)	<0.001	2.718 (1.624–4.550)	<0.001	0.795 (0.540–1.170)	0.245	0.831 (0.568–1.216)	0.340
Company employee	2.325 (1.301–4.155)	0.004	2.535 (1.439–4.465)	0.001	0.788 (0.513–1.210)	0.275	0.799 (0.522–1.221)	0.299
Education								
Secondary School or below (Reference)	1	–	1	–	1	–	1	–
Masters/Bachelor degree	0.657 (0.350–1.232)	0.190	0.667 (0.381–1.169)	0.157	0.596 (0.398–0.892)	0.012	0.520 (0.358–0.756)	0.001
Associate degree	1.884 (1.168–3.037)	0.009	1.977 (1.245–3.139)	0.004	0.738 (0.506–1.077)	0.115	0.723 (0.499–1.047)	0.086

Multivariable logistic regression analysis was performed to determine the independent correlation between TTI status and demographic factors, and suggested that TTIs status had a curvilinear relationship with age, previous donation history, marital status, occupation, and education (*p* < 0.05) (Table 3). In addition, blood donors who were over 26 years old were more likely infected with TTIs (age [26–35], aOR: 2.799, 95% CI: 1.948–4.023; age [36–45], OR: 2.562, 95% CI: 1.862–3.525; age 46–55, OR: 1.813, 95% CI: 1.376–2.389; all *p* < 0.001). Married subjects (aOR: 0.720, 95% CI: 0.573–0.904, *p* = 0.005), repeat donors (aOR: 0.261, 95% CI: 0.204–0.334, *p* < 0.001), who were teachers/civil servants/medical workers (aOR: 0.355, 95% CI: 0.181–0.694, *p* = 0.002), and had higher education (masters/bachelor degree) (aOR: 0.602, 95% CI: 0.467–0.775, *p* < 0.001) had lower probability of acquiring TTIs.

With regard to each TTI, age and previous donation history were independent predictors of HBV, HCV, and syphilis infections, but not HIV (*p* < 0.05), and only syphilis infection was related to ethnicity (aOR: 2.309, 95% CI: 1.378–3.868, *p* = 0.001). Among all TTIs, only HBV positivity was not associated with marital status (aOR: 0.933, 95% CI: 0.670–1.299, *p* = 0.681) and HCV infection was not related with occupation (*p* > 0.05). Furthermore, gender (HIV, aOR: 10.393, 95% CI: 4.193–25.762, *p* < 0.001; syphilis, aOR: 0.532, 95% CI: 0.406–0.697, *p* < 0.001), and education (HIV: associate degree, aOR: 1.977, 95% CI: 1.245–3.139, *p* = 0.004; masters/bachelor degree, aOR: 0.520, 95% CI: 0.358–0.756, *p* < 0.001) were independent predictors of HIV and syphilis infections (Tables 4 and 5).

## Discussion

This multicenter study investigated the demographic features including age, gender, previous donation history, ethnicity, marital status, occupation, and education and determined their association with TTIs, as well as potential risks of TTIs among blood donors in 14 different blood centers/blood banks in China during March 2015 and September 2015. The samples that tested non-reactive for TTIs (ALT > 50 U/L) were enrolled in the study as TTI negative controls that showed the high agreement of demographic distribution with normal voluntary blood donors, according to the China Report on Blood Safety 2016 [3].

From the demographic characteristics of 1976 blood donors, most donors were married males, aged between 18 and 45 years and were mainly first-time donors. These donors were workers or company employees mainly of Han ethnicity and had educational levels of secondary school or below. In summary, our findings suggested that the proportion of populations aged between 26 and 55 years, first-time donors, and less-educated people among the TTI groups were significantly higher than those that tested TTI negative, which was similar to the findings of other studies on the demographic characteristics associated with TTIs in China [4, 5]. According to bivariate logistic regression analyses, our findings indicated that age, previous donation history, marital status, occupation, and education were correlated with TTIs status. All the demographic factors mentioned above were independent predictors for TTIs. Specifically, younger blood donors who were married and repeat donors with high educational level were less likely to be infected with TTIs. Besides, donors who were teachers, civil servants, or medical workers were less susceptible to TTIs. Large floating populations, mainly workers and company staffs who are principally unmarried males, sexually active, and far from their families are susceptible to TTIs [6].

However, different kinds of TTIs vary from each other regarding socio-demographic information. Only demographic characteristics of HBV infected individuals were most similar to the overall TTI population. For HCV infection, all socio-demographic features but occupation were parallel to TTIs. The most special characteristics were found in TP and HIV infection. All the demographic factors of TP positives significantly varied from the negatives and were associated with TP infection. Specifically, the proportion of donors aged over 36 years was apparently higher among those with HBV, HCV and HIV, and had a higher risk of TP infection. The same phenomenon was observed in another study conducted in China [5] on lifelong TP antibody persistence after syphilis infection; hence, the proportions of donors with TP serological reactivity were positively related to age. The proportion of female donors among TP positives (44.9%) was consistent with 2008–2010 data reported by Jing Liu et al. [7]. According to the transmission route of syphilis, the probability of TP infection is higher among females than males through heterosexual transmission; besides, the rather low clinical visiting rate of females with TP reactivity leads to rapid spread due to highly concealed symptoms, in comparison to males [8]. Regarding ethnicity, only TP positive subjects had obviously higher proportions in minority populations, which largely agreed with a study conducted in 2013 [9]. Therefore, syphilis prevention and control should be initiated among these blood donors. Marital status and gender were independent predictors of HIV infection. In the HIV positive groups, unmarried male donors occupied a higher proportion than non-reactive subjects for TTIs (ALT> 50 U/L) and the other three TTIs. Furthermore, the proportion of teenagers (18–25 years) with HIV infection was significantly higher than among those with HBV, HCV, and TP. Regarding occupation, the findings indicated that HIV infected donors occupied the highest percentage, compared with TTI negative subjects and those with the other TTIs, which was similar to previous studies [10, 11]. The majority of workers were unmarried young men, sexually active and far from families [12] who might be prone to active involvement in extramarital sex, including sex with commercial sex workers [13]. With lower educational levels, they are generally unaware of HIV/AIDS [13, 14]. Owing to epidemiological, behavioral, and social circumstances, workers are more susceptible to HIV/AIDS compared to the general population [15, 16] and may contribute to the spread of HIV in China [17].

In addition to the promotional effect of workers and company employees in the spread of HIV, the dramatic accumulation of HIV transmission among men who have sex with men (MSM) in recent years should also be taken into consideration [18]. The peculiar sexual behaviors among MSM put their sexual partners at high risk of HIV infection due to easy rectal mucosal damage [19]. Apart from workers, the HIV positivity rate among students was also high in our study, and all HIV infected students were males. A recent meta-analysis showed that students who are MSM are becoming a high-risk population for HIV infection in China [20]. To make things worse, the infection rate is high but the rate of condom usage remains low among MSM [13]. Regarding previous donation history, all TTIs apart from HIV showed significantly different proportions compared to non-reactive individuals. Normally, the proportion of repeat donors among TTIs negatives is evidently higher than among TTI populations [2, 9]. However, blood donors with high risk behavior may donate blood deliberately and repeatedly to determine whether they are infected with HIV. Furthermore, blood donors in China have different definitions for first-time and repeat blood donors, and there is no good info-relation between blood donation systems in various cities and even in different regions. For instance, a blood donor who donates blood repeatedly in the same blood center might still be recognized as a first-time donor in another blood center. Hence, the number of first-time donors may actually be overestimated. Strengthening identification information networking with other blood centers, hospitals and the Centers for Disease Control, throughout China can effectively differentiate first-time and repeat blood donors and defer high-risk behavior or clinical symptoms of blood donors.

### Limitations

The limitations of this study were as follows: 1) The study period was relatively short, and the sample size was inadequate. 2) Information on TTI risk behaviors such as intravenous drug use, MSM, and commercial sexual encounters should be included in this study to confirm the association of high-risk factors with TTIs status among blood donors.

## Conclusion

The present study suggests that age, gender, previous donation history, ethnicity, marital status, occupation and education may have associations with each TTI. The majority of susceptible populations for TTIs are unmarried males and first-time donors aged between 26 and 55 years. Besides, blood donors who are workers or company employees with low education level may be more likely to acquire TTIs. Our findings remind us of the critical essence of timely surveillance as well as updated demographic data on certain high-risk populations such as workers and company staffs among blood donors. Moreover, the urgent need for blood safety indicates we must popularize the health examination requirements for blood donors, effectively raise awareness of TTIs through strong policy and economic support, strengthen the antiviral therapeutic strategy of TTIs, and fulfill comprehensive TTI control programs in China.

## Abbreviations

HBV: hepatitis B virus
HCV: hepatitis C virus
HIV: human immunodeficiency virus
ID-NAT: individual donation-nucleic acid testing
NCCL: National Center for Clinical Laboratories
RIBA: recombinant immunoblot assay
TP: *Treponema pallidum*
TTIs: transfusion transmitted infections
WB: Western Blot

## References

[1] Global Status Report on Blood Safety and Availability 2016 [https://apps.who.int/iris/bitstream/handle/10665/254987/9789241565431-eng.pdf?sequence=1].

[2] Li C, Xiao X, Yin H, He M, Li J, Dai Y, Fu Y, Ge J, Yang Y, Luan Y: Prevalence and prevalence trends of transfusion transmissible infections among blood donors at four chinese regional blood centers between 2000 and 2010. *Journal of Translational Medicine,10,1(*2012*-08-28)* 2012, 10(1):176. 10.1186/1479-5876-10-176 PMC3493321 22929614

[3] China’s report on blood safety 2016. Beijing: PEOPLE'S MEDICAL PUBLISHING HOUSE; 2017.

[4] Zaller N, Nelson KE, Ness P, Wen G, Kewir T, Bai X, Shan H (2006). Demographic characteristics and risks for transfusion-transmissible infection among blood donors in Xinjiang autonomous region, People's Republic of China. Transfusion.

[5] Yang S, Jiao D, Liu C, Lv M, Li S, Chen Z, Deng Y, Zhao Y, Li J (2016). Seroprevalence of human immunodeficiency virus, hepatitis B and C viruses, and Treponema pallidum infections among blood donors at Shiyan, Central China. BMC Infect Dis.

[6] Sumita M (2011). Communiqué of the National Bureau of statistics of People's Republic of China on major figures of the 2010 population census (no.1). China Population Today.

[7] Liu J, Huang Y, Wang J, Guo N, Li J, Dong X, Ma H, Tiemuer M, Huang M, Wright DJ (2012). The increasing prevalence of serologic markers for syphilis among Chinese blood donors in 2008 through 2010 during a syphilis epidemic. Transfusion.

[8] Tucker JD, Cohen MS (2011). China’s syphilis epidemic: epidemiology, proximate determinants of spread, and control responses. Curr Opin Infect Dis.

[9] Pan X, Zhu Y, Wang Q, Zheng H, Chen X, Su J, Peng Z, Yu R, Wang N (2013). Prevalence of HIV, syphilis, HCV and their high risk behaviors among migrant workers in eastern China. PLoS One.

[10] Wang J, Liu J, Huang Y, Yang T, Yao F, Dong X, Wen G, Bi X, Zhao M, Wen X (2013). An analysis of risk factors for human immunodeficiency virus infection among Chinese blood donors. Transfusion.

[11] Zeng P, Liu Y, He M, Wang J, Keating S, Mao W, Huang M, Ma H, He W, Bi X (2017). The infection staging and profile of genotypic distribution and drug resistance mutation among the human immunodeficiency virus-1 infected blood donors from five Chinese blood centers, 2012-2014. PLoS One.

[12] Sumita M (2011). Communiqué of the National Bureau of statistics of People's Republic of China on major figures of the 2010 population census (no.2). China Population Today.

[13] Yang B, Wu Z, Schimmele CM, Li S (2015). HIV knowledge among male labor migrants in China. BMC Public Health.

[14] Qin QQ, Guo W, Wang LY, Ding ZW, Cai C, Cui Y, Sun JP (2016). The characteristics of HIV-positive men who have sex with men in China and predictors of their migration, 2008-2015. Zhonghua yu fang yi xue za zhi [Chinese journal of preventive medicine].

[15] Saggurti N, Verma RK, Jain A, RamaRao S, Kumar KA, Subbiah A, Modugu HR, Halli S, Bharat S (2008). HIV risk behaviours among contracted and non-contracted male migrant workers in India: potential role of labour contractors and contractual systems in HIV prevention. Aids.

[16] Aung E, Blondell SJ, Durham J (2017). Interventions for increasing HIV testing uptake in migrants: a systematic review of evidence. AIDS Behav.

[17] Zheng N, Guo Y, Padmadas S, Wang B, Wu Z (2014). The increase of sexually transmitted infections calls for simultaneous preventive intervention for more effectively containing HIV epidemics in China. BJOG : an international journal of obstetrics and gynaecology.

[18] 2015 China AIDS Response Progress Report [http://www.unaids.org/sites/default/files/country/documents/CHN_narrative_report_2015.pdf].

[19] Pescatore NA, Pollak R, Kraft CS, Mulle JG, Kelley CF (2018). Short communication: anatomic site of sampling and the rectal mucosal microbiota in HIV negative men who have sex with men engaging in Condomless receptive anal intercourse. AIDS Res Hum Retrovir.

[20] Li Y, Xu J, Reilly KH, Zhang J, Wei H, Jiang Y, Geng W, Tang W, Shang H (2013). Prevalence of HIV and syphilis infection among high school and college student MSM in China: a systematic review and meta-analysis. PLoS One.

